# Barriers and facilitators of colorectal cancer screening in Asia

**DOI:** 10.3332/ecancer.2021.1285

**Published:** 2021-09-13

**Authors:** Sare Hatamian, Fatemeh Hadavandsiri, Zohre Momenimovahed, Hamid Salehiniya

**Affiliations:** 1 Department of Epidemiology, School of Public Health and Safety, Iran University of Medical Sciences, Tehran, Iran; 2 Cancer Research Center, Shahid Beheshti University of Medical Sciences, Tehran, Iran; 3 Department of Midwifery and Reproductive Health, Qom University of Medical Sciences, Qom, Iran; 4 Social Determinants of Health Research Center, Birjand University of Medical Sciences, Birjand, Iran

**Keywords:** colorectal cancer, screening, facilitators, barriers, Asia

## Abstract

**Purpose:**

One of the most common cancers in Asia is colorectal cancer (CRC). Early diagnosis and timely treatment are necessary for preventing complications and advanced stages of the disease. It is important to evaluate barriers and facilitators of screening in different countries. This systematic review aimed to identify the barriers and facilitators of CRC screening in Asia.

**Methods:**

In this systematic review, for identifying barriers and facilitators of CRC screening, a comprehensive search was conducted in PubMed, Web of Science and Scopus in 12 December 2020. Combination keywords such as colorectal cancer, screening, sigmoidoscopy, colonoscopy, faecal occult blood test, barriers, facilitators and the names of each Asian country were used for searching. Full text original studies in English language were accepted in the review.

**Results:**

In total, 36 articles were included in the review. Barriers and facilitators were evaluated. The most common reported barriers were lack of knowledge, fear of result, fear of procedure, fear of pain, lack of awareness, high cost and lack of gastrointestinal symptoms. The most frequent facilitators were having knowledge and awareness of CRC screening, perceived risk and severity, family history of cancer and physician recommendation.

**Conclusion:**

For promoting success in CRC screening programmes, knowing what the barriers and facilitators are is necessary. Awareness and various personal, professional and social factors have been shown to be the major barriers toward CRC screening in most Asian countries.

## Introduction

Cancer is recognised as a global problem nowadays. Colorectal cancer (CRC) is ranked as the third most common cancer in the world by International Agency for Research on Cancer which reported 0.8 million deaths related to CRC in 2018 [1]. It is estimated by the year 2030, the worldwide burden of CRC will rise by 60% to more than 2.2 million new cases and 1.1 million deaths [2, 3]. In Asia, a high prevalence and an increasing number of CRC in both genders have been reported [4, 5].

Due to the high prevalence and incidence of CRC, early diagnosis and timely treatment are necessary for preventing complications and advanced stages of disease. With prevention, 40% of cancers can be prevented and by early detection 90% of cancers can be treated [6–8]. Results of previous studies show that by timely screening in CRC, 100% of genetic cases can be prevented [9, 10]. For early diagnosis of CRC, regular screening is the best control measure and effective method [11–13].

As people become more aware of the risk factors for CRC, their participation in screening programmes increases. Factors leading to CRC are increasing age, life style, family history of CRC, smoking, alcoholism, a low fibre diet, red and processed meat consumption [14–16]. Lack of public knowledge about risk factors of CRC leads to development of disease [14]. The United States Preventive Services Taskforce recommends colorectal screening methods such as: Faecal occult blood test (FOBT), as the simplest way of screening that should be done every year, sigmoidoscopy is done once every 5 years and colonoscopy done at least every 10 years in older than 40-year-old participants [17, 18].

Screening programmes are challenging in developing countries; programmes need huge allocations of financial and logistic resources. Before intending for screening projects, financial and individual factors such as knowledge, attitude, awareness and belief of health promotion should be considered [11, 19–25]. Due to the importance of knowing the causes of participation-status in screening, this study was conducted to determine the barriers and facilitators of colorectal screening programme in Asia.

## Materials and methods

### Search strategy

For this systematic review which was designed in 2020, comprehensive search was conducted in PubMed/Medline, Web of Science and Scopus in December 2020. Combined keywords such as colorectal cancer, screening, sigmoidoscopy, colonoscopy, faecal occult blood test (FOBT, barriers, facilitators and names of each Asian country were used for searching. We used manual searches in valid journals and followed articles and full text articles for comprehensive search. The articles were entered to EndNote and duplicate articles were deleted automatically by EndNote-X8 software. After removing duplicates, a screening of titles and abstracts was performed and eligible articles were selected. Full-text articles were then reviewed and articles that determined barriers and facilitators of CRC screening were included.

### Inclusion criteria

In this study, inclusion criteria were being an original article, observational studies (cross-sectional studies, case–control and longitudinal cohort studies) that investigated CRC screening barriers and facilitators, referring to CRC screening modalities and factors, using keywords in their title or abstracts.

### Exclusion criteria

Articles such as letters to editor, case reports, conference abstracts, editorials, review studies, clinical trials and studies not having the full text were the exclusion criteria.

### Data selection and synthesis

Searching the article was done by one of the researchers, two researchers’ evaluated articles by prepared checklist for data extraction. After excluding irrelevant articles, the full text of remaining studies was reviewed. Extracting the results was done qualitatively. Information was extracted from each study: the first author’s last name, year of study, study location, type of statistical analysis (descriptive, analytic), type of cancer screening, study population, study objectives and main findings.

### Qualitative assessment and analysis

For quality assessment of included studies, we used NewCastle Ottawa Quality Assessment Scale for quantitative studies. The tool uses eight items, categorised into three groups: selection, comparability and ascertainment of either exposure or outcome. Numbers showing awarded for each quality items as visual assessment. No studies were excluded based on their quality score [26].

## Results

### Specification of included studies

**Figure 1.**
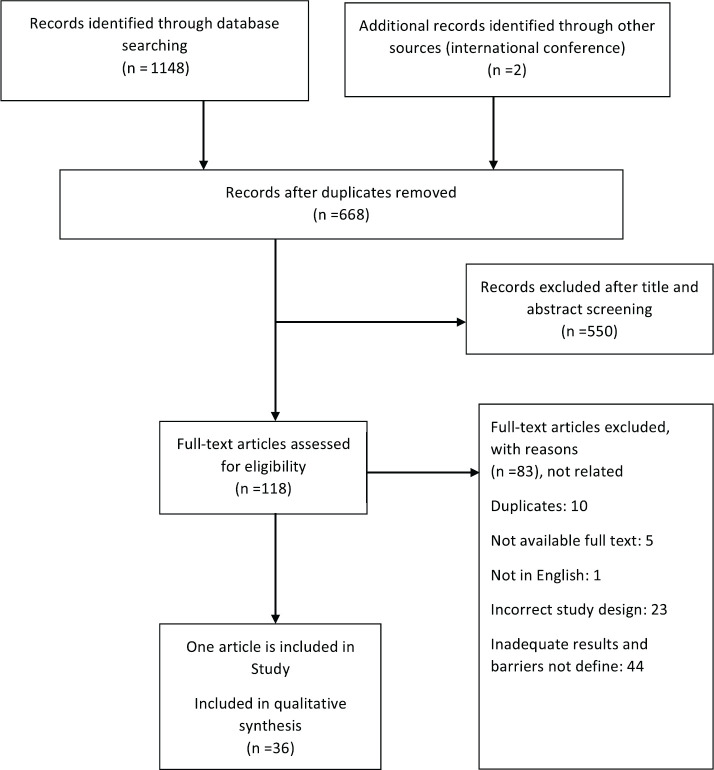
PRISMA (Preferred Reporting Items for Systematic Reviews and Meta-Analyses) flowchart illustrating the process for the selection of the included articles for the systematic review.

Total search of databases determined 1,150 studies; 482 studies were excluded because of duplication. After checking the title and abstract, 550 studies were excluded that were not related to the purpose of review article and its criteria. Besides, full text screening was done on 118 studies, and finally 36 articles were accepted for this systematic study (Figure 1).

### Study characteristics

Basic characteristics of the included studies in this review are presented in Tables 1 and 2. Studies selected for literature were cross-sectional studies. The number of samples in studies varied from 116 to 7,200. Most of the participants were 40 years old and the majority of them were men.

### Study quality assessment

Scores for cross-sectional studies ranged 5–7 by Ottawa scale. Samples of studies were representative of target population in 30 studies. Data collection procedures were described well by all of studies. Reported studies using self-administered questionnaire and some of them using health belief model (HBM) questionnaire. A total of 36 studies were used sufficient analysis methods and analysis linkage. A total of 26 studies had good quality score, and 10 studies had fair quality score by Ottawa quality scale (Table 2).

Barriers and facilitators are shown in Table 3.

### Barriers and facilitators

#### Knowledge of screening

In any screening programme, especially CRC screening, knowledge and awareness are considered as a crucial element. Knowledge of risk factors and screening methods leads to increased use of screening [12, 28, 30]. General lack of knowledge of CRC screening methods and risk factors were known as barriers in five studies of different countries [20, 22, 28, 34, 47]. Althobaiti and Jradi [28] showed that low participation in screening is related to lack of knowledge of screening and symptoms of CRC. Low level of knowledge stems from low level of education in relation to low level of awareness and attitudes. Another study among older Saudis showed that prior information about signs and risk factors had positive influence on awareness and intention to screening [29]. In a study in United Arab Emirates, overall evaluation of knowledge revealed a poor level of knowledge on risk factors, and only 40% of adults identified FOBT as a main screening test for CRC prevention [12].

In a study in China, individuals who have knowledge of screening tests were six times more likely to perform CRC screening rather than those who do not have any knowledge (high: AOR: 6.68, 95% CI = (4.36, 10.24), *p* < 0.001) [15]. Positive attitude that screening can be effective in early detection and reducing treatment time leads to decision to participate in CRC screening. Results of three studies demonstrate that when persons are aware of signs and risk factors of CRC, their participation in CRC screening increases [12, 29, 51]. According to Tfaily *et al* [38] study in Lebanon, people with higher awareness of risk factors were 2.2 times more likely to participate in CRC screening (OR = 2.221, 95% CI = (1.023, 4.820), *p*-value = 0.04). Believing that CRC is preventable is about (73.3%) and curable (70.5%) effected on CRC screening two times more strongly for choosing FOBT method as test (OR = 2, 95% CI: 1.04–2.29) [21].

#### Perceived severity, seriousness, barriers, risk, susceptibility, benefit

Other motivators of participation in CRC screening are perceived risk, severity and seriousness barriers. In many studies, results showed that perceived severity, seriousness and susceptibility leads to screening, and results of perceived barriers had a negative effect on screening [18, 42, 46, 48, 50–52]. In a study, perception towards sub scales and health motivators was seen. Results showed that there was a significant positive correlation between knowledge of CRC screening and perceived susceptibility, seriousness and perceived barriers. Knowledge of CRC screening has a greater effect on perceived susceptibility to CRC, the seriousness of CRC, barriers for CRC and health motivation than those without knowledge about it [46, 52]. Participants who perceived fewer barriers (OR = 0.37; 95% CI: 0.21–0.89), perceived more susceptibility (OR = 2.99; 95% CI: 1.23–5.45) were more likely to utilise screening tests [50]. Studies showed that some facilitators such as knowledge, awareness, sociodemographic factors, self-efficacy, perceived barriers, susceptibility, severity and benefits had positive influence on CRC screening [12, 20, 27, 28].

Higher self-efficacy intent for screening was 1.14 times higher in participants (OR = 1.14, 95% CI: 1.04–1.26) [45].

#### Fears of result, fear of procedure

Common psychological barriers have been shown such as fear, embarrassment, anxiety and pain in most studies [20, 22, 31, 37–39, 41–43]. Results of five studies showed fear of the painful medical procedures [11, 20, 38, 39, 41]. Fear of tumour detection and test result subsequent fear of developing

CRC and fear of complications cause ignorance of screening [11, 22, 29, 33, 41, 43, 46, 49]. Of the included studies, 51.6% reported fear of painful medical procedures as perceived barriers [11]. Procedure of screening may be embarrassment for participants. Al-naggar *et al* [22] showed that participants did not want to do screening, because of shyness (55.1%), painful procedure in FOBT (53.5%), embarrassment (55.1%) in sigmoidoscopy and then 32.1% fear of cancer detection.

#### Professional factors

After patient related barriers, professional factors as healthcare system barriers were categorised as common barriers. These barriers include the following: lack of recommendation by doctor or medical health staff [12, 20, 22, 23, 28, 31, 34, 35, 46, 48], lack of integrated and updated guidelines in health care centres [28, 31, 37, 42], lack of resources [12, 28].

According to Althobaiti and Jradi’s [28] study, it was described that among medical students, knowledge of CRC factors and screening modalities was poor (52.47% and 57.83%, respectively). On the other hand, increase in medical education increased knowledge of screening three-fold (OR = 3.23; 95% CI: 2.01–5.18) and attitudes toward low level of medical science education were increased two (OR = 2.74; 95% CI: 1.86–4.03) times higher [28].

Results of Chen’s study [31] showed that majority of physicians’ barriers toward CRC were identified as lack of knowledge of colorectal guidelines (46.7%) and lack of sufficient information about CRC patients for early screening (43.8%). A study in Singapore on motivators such as presence of symptoms (92%), physician’s recommendation (81.4%) and family history (70.7%) reported increased screening. Physicians recommendation had 7.15 times higher influence (OR = 7.10 (95% CI: 3.08–16.4), *p* < 0.001) on screening among survivors [17]. Recommendation by a doctor has a positive effect on screening, while believing that the screening process is painful has a negative effect on screening. The results of a study show that 95% of people report lack of doctor’s advice as a barrier to screening [12, 49]. Physician recommendations and advice [12, 37, 40], promoting knowledge and awareness of medical staff and students, reconciling guidelines of CRC screening are the strong motivating factors of CRC screening in different studies [24, 31, 34, 37, 48]. In a study among Saudi patients, 43% of the participants got knowledge of screening by regular awareness programmes from health care system [11].

#### Costs of screening

Medical costs associated with screening were a barrier in six studies. Huang *et al* [17] reported that cost of screening is too expensive and caused 50% of barrier of screening.

#### Time constraints

Lack of time [12, 20, 21, 36, 41, 43], accessibility to CRC screening were illustrated as more barriers reported by studies [17, 31, 32, 36, 43, 49, 51].

Long waiting times in public hospitals is one of the barriers in Saudi Arabia, Korea and Malaysia [12, 36, 41, 43].

#### Accessibility

One of the important barriers for screening was about accessibility, lack of transportation and screening availability which differ from area residency in a country. More barriers have been reported from participants who live in rural areas [20]. In a study in Saudi Arabia, general lack of unavailability of FOBT was the only important barrier of CRC screening [20].

#### Socio demographic factors

Demographic specifications influenced the use of screening modalities and affected on barriers and facilitators. Characteristics such as age [12, 29, 32, 36–39, 46, 48, 51], gender [12, 20, 22, 27, 29, 32, 45, 47, 48], level of education [11, 12, 14, 23, 28–30, 32, 37, 39, 40, 47, 51], socioeconomic status and employment status [12, 17, 23, 37], marital status [29], ethnicity [12, 48] have been examined.

In a study in Singapore, younger participants (OR = 3.21, 95% CI: 1.01–5.41, *p* < 0.01) and more educated (OR = 1.54, 95% CI: 0.48–2.61), *p* < 0.01) had the highest rate of screening [51]. In a study of Al-Hajeili *et al* [14], level of education (*p* = 0.001) and region of residence (*p* = 0.02) significantly associated with knowledge of screening, knowledge about colonoscopy was associated with gender (*p* = 0.03), educational level (*p* < 0.01) and family history of CRC (*p* = 0.04). According to the study by Alhuzaim *et al* [11], the level of education has a positive role in the knowledge, behavior and self-efficacy of the participants. In this study, 65% of educated people are more inclined to be screened.

According to this study, increased age > 50 and level of education below secondary school were associated with decreased odds of CRC screening, odds ratio of age 0.9 (OR = 0.9, 95% CI: 0.50–0.99, *p* = 0.002) indicated low CRC screening than younger participants and about educational level 0.7 (OR = 0.7, 95% CI: 0.53–0.95, *p* = 0.02) below secondary school had lower CRC screening compared with high level of education [37]. On the other hand, study of Tfaily *et al* [38] demonstrated that older participants (above 50 years of age) had two times more knowledge and 55% awareness about CRC screening and 43% willingness to do screening. The study of Galal *et al* [29] showed that gender, unmarried and having less than college education were considered reducing predictors of CRC screening. Unmarried participants had 0.11 times lower CRC screening-rate (OR = 0.11; 95% CI: 0.10–0.23; *p* = 0.001) than married participants for screening. In a study among adults in United Arabs Emirates, knowledge of participations between UAE nationals and non-UAE nationals had significantly differences (*p* < 0.001), non-UAE nationals had better knowledge [12].

People over the age of 50 were more aware of the signs and symptoms than other participants in the study [38]. One of the important motivators that influences CRC screening is self-efficacy [11, 45]. In a study in Iran, self-efficacy (OR = 1.17, 95% CI: 1.08–1.27) plays a role as a motivator variable about CRC screening among other participants [45].

#### Lack of signs and symptoms

One of the barriers of screening CRC reported by participants included lack of symptoms. Six studies reported that participants with no symptoms lead to lower screening history of symptoms and believed sickness caused more participation in screening [3, 4, 20, 22–23, 29–31]. In a study in Saudi Arabia, 73.4% participants reported absence of signs and symptoms as the most important barrier [20].

#### Social factors and communications

Family and friends and relatives’ recommendations have a role in raising sufficient awareness.

History of cancer in family members motivated others for screening by increasing knowledge of screening [11, 14,17, 29, 31, 40, 46, 48, 49]. A Saudi-Arabian study having relatives diagnosed with CRC screening (OR = 1.67, 95% CI: 0.99–2.81, *p*-value < 0.0001) leads to believing in the effect of screening to detect cancer [14].

Media and social networks, physician’s recommendations are the main sources of encouragement [11, 12, 17, 21, 23, 29, 38, 45, 49, 50]. On the other hand, a study showed that risk groups having positive family history of CRC, screening did not have increased clinical knowledge and awareness [23]. Awareness of CRC screening was two times higher in participants with clinical recommendation (OR = 2.384, 95% CI = (1.20–4.70), and *p*-value = 0.012), and those who undergo regular physician check-ups have three times higher (OR = 3.167, 95% CI = (1.88–5.32), *p*-value < 0.0001) level of awareness [38].

Ways reported to increase knowledge and information were through media (such as books, newspapers, magazines, TV, radio, Internet, knowledge from health care staff, family members and friends information) [23, 24, 29, 44, 45, 48, 49, 51, 52].

### Comparisons of countries

Studies from Saudi Arabia and Palestine demonstrated that one of most common barriers was lack of physician recommendation, absence of signs and symptoms and lack of knowledge of CRC [11, 20, 21, 30, 37]. In the eastern region of Saudi Arabia, lack of provider’s knowledge of recommended screening and lack of public awareness of CRC screening were most common barriers [29]. Financial problems had no effect on participating in CRC

screening, because a large population had access to free screening tests that covered by the ministry of health [11, 20, 21, 29, 30, 40, 53]. In south East of Asia, Malaysia and Singapore, fear of cancer, avoiding doing screening after lack of knowledge, lack of recommendation by physician were the most common barriers of CRC screening [22, 34, 35, 43, 46, 48, 49]. In a study from Iran, more than 90% population did not have any knowledge of CRC risk factors, symptoms and screening tests [23]. The rate of FOBT screening was 29.9% [24]. Lack of awareness and limited literacy, lack of physician recommendation were as the most common barriers [23, 24]. In South Korea, only 31.7% of target population participated in screening programmes and one of the barriers was cost of screening that most of the cost paid by participants [41].

## Discussion

Our systematic review found a literature evaluating barriers and facilitators to CRC screening of participants from cross-sectional studies from Asia. Different modalities of CRC screening such as FOBT, colonoscopy, sigmoidoscopy have been introduced for diagnosing CRC in different countries of Asia for many years. Reports demonstrated that in many countries doing CRC screenings, there is a poor level and score of knowledge, and attitude of doing screening is low. More awareness and recommendations about screening tests are needed, as well as further investigation. Our study compared Asian countries for different barriers of CRC screening.

This review of quantitative studies is relevant to the general population, physicians and medical students who are providing CRC screening need to be promoted about CRC screening factors. Results of 36 studies demonstrated that factors influencing the decision to participate in CRC screening are knowledge, attitude of CRC as a curable disease and lack of knowledge about CRC and CRC screening modalities. Knowledge was revealed as important point relating to participating in screening. Increased knowledge had a positive impact on attitude toward CRC, then had a stronger intent for screening. Obviously factors such as education level, cultural and social barriers affect on searching for CRC risk factors and then tendency to CRC screening. Using information sources such as media, videos, books and physician recommendation was found to have an impact on CRC screening. On the other hand, the main factors for ignoring the screening are the lack of knowledge, cost, fear of diagnosis, fear of screening procedures, lack of time, embarrassment. Remarkably, the difference in facilitators and barriers results in different groups with different sociodemographic factors and different guidelines which use the maximum reported score of doing screening modalities is 73% and the lowest is 0.7%. Information on factors on CRC screening such as knowledge, attitude and barriers are poor and need to be further considered, raising knowledge and awareness are equal for reducing barriers. However, appropriate guidelines and protocols must be developed.

Lack of integrated guidelines in countries and low level of knowledge among medical students are also common barriers. Asian population had a poor knowledge-rate and low rate of screening in comparison to western and American societies [54, 55]. On the other hand, media and social communications and family history of cancer, physician recommendation played an effective role on screening-participation. The most common barriers in Asian countries were lack of knowledge, lack of physician recommendation and fear of screening. In comparison to western countries’ fear of screening results and fear of screening procedure, in American countries cost of screening was the most common barrier of screening. Physician recommendation in Asian countries was low in contrast to the American physician recommendation was 72.6% [47, 55].

### Strength and limitations of the review

Our search strategy was inclusive and we searched a wide range of databases and then to enhance sensitivity we retrieved full text of all selected articles. We included studies with a large number of participants, who intended to evaluate screening barriers and facilitators. We compared barriers and facilitators from different regions of Asia.

In this review, we compared countries and demonstrated barriers and facilitators of each studies. A limitation was that we used quantitative studies and we recommended to use both qualitative and quantitative studies.

## Conclusion

We found that lack of knowledge and awareness about CRC and CRC screening was preventing participation in CRC screening in Asia. While interventional education and guidelines are concurrent with logistics, cultural and motivational barriers must be overcome for reducing inequities in participation in CRC screening. Awareness programmes by health care officials, governments and health care organisations can lead to increased knowledge and ultimately to regular participation in screening. Our study showed that Asian countries have similar barriers and facilitators.

## Funding

Not applicable.

## Conflicts of interest

None.

## Availability of data

Not applicable.

## Ethics approval

Not applicable.

## Consent to participate

Not applicable.

## Figures and Tables

**Table 1.** Characteristics of included studies in the review.

Study characteristics	No (%) of the studies (*n* = 36)
Year 2005–2009 [51] 2010–2014 [23, 30, 31, 35–37, 39, 41, 43, 46, 48, 49, 52] 2015–2020 [11, 12, 17, 18, 20–22, 24, 27–29, 32–34, 38, 40, 44, 45, 47, 50, 53]	1 (2.7) 13 (38.8) 22 (58.3)
Participation number 100–<200 [17, 18, 22, 34, 39, 42, 43] 200–<500 [20, 30, 32, 33, 36, 38, 45, 46, 50, 52, 53] 500–<1,000 [11, 12, 21, 24, 28, 29, 31, 40, 48, 51] >1,000 [23, 27, 35, 37, 41, 44, 47, 49]	7 (19.4) 11 (30.5) 10 (27.7) 8 (22.2)
Type of quantitative studies Cross-sectional [11, 12, 17, 18, 20–24, 27, 28, 29–53] Cohort [47, 48]	34 (94.4) 2 (5.5)
Country United Arab Emirates [12] Saudi Arabia [11, 20, 21, 27–30, 40, 53] Malaysia [22, 34, 35, 43, 46, 48] Iran [23, 24, 44, 45, 50] China [18, 31, 47] Pakistan [32, 33] Singapore [17, 49, 51] Thailand [36] Palestine [37] Lebanon [38] Turkey [39] Korea [41] Jordan [42, 52]	1 (2.7) 9 (25) 6 (16.6) 5 (13.8) 3 (8.3) 2 (5.5) 3 (8.3) 1 (2.7) 1 (2.7) 1 (2.7) 1 (2.7) 1 (2.7) 2 (5.5)
Screening method FOBT [11, 20, 24, 28, 29, 44, 45, 47, 51, 52] Colonoscopy [12, 18, 20, 21, 28, 44, 47, 52] Various methods (FOBT, colonoscopy, sigmoidoscopy and FIT) [17, 22, 23, 27, 32, 37–41, 48, 49] NA [30, 31, 33–36, 42, 43, 46, 50, 53]	11 (30.5) 8 (22.2) 13 (38.8) 9 (25)

FIT: fecal immunochemical test; NA: not applicable

**Table 2.** Characteristics of included studies in the review.

Study	Study location	Design	Age	Sample size/gender	Screening type	Statistical analysis	Type of questionnaire/type of samples	Quality score^a^	Facilitators Barriers
Al Abdouli *et a*l [12]	United Arab Emirates/ Western Asia	Cross-sectional survey	29–50	600 251 male	Colonoscopy	Descriptive analysis	A structured bilingual questionnaire in English and Arabic/healthy population	Fair	Positive attitude towards screening, age, gender, educational level and occupation related significantly. Towards knowledge education is significant. Practice: education, occupation
Alduraywish *et al* [20]	Saudi Arabia Western Asia Urban and rural	Cross-sectional survey	45–66	448 215 male	Colonoscopy FOBT	Descriptive analysis	Self-administered questionnaire or interview/Patient population from hospital	Good	Barriers Gender, residency area (living in rural), history of CRC screening, lack of knowledge about CRC, absence of symptoms and signs, fear of results had significantly related to barriers undergone screening
Almadi *et al* [21]	Saudi Arabia Western Asia	Cross-sectional survey	18–27	500 250 male	Colonoscopy	Descriptive analysis and multivariate	Questionnaire based on HBM/Health population	Good	Facilitator Age significantly associated to willing screening
Almadi *et al* [27]	Saudi Arabia Western Asia	National wide survey	20–70	5,720 4,091 male	Various methods (FOBT, colonoscopy and sigmoidoscopy)	Descriptive analysis and multivariate	Questionnaire based on HBM/Health population from different region of urbans	Good	Facilitator Gender significantly associated to willing screening
Al-Naggar *et al* [22]	Malaysia Southeast Asia	Cross-sectional survey	>50	187 93 male	Various methods (FOBT, colonoscopy and sigmoidoscopy)	Descriptive analysis and multivariate	Self-administered questionnaire or interview /samples from hospital	Fair	Facilitator Age, gender, income, occupation had significant relation towards knowledge, attitude and practice
Althobaiti and Jradi [28]	Saudi Arabia Western Asia	Cross-sectional survey	<22, >23	581 278 male	FOBT, colonoscopy	Descriptive analysis and multivariate	Self-administered questionnaire or interview/medical students	Fair	Facilitator Knowledge Age OR = 2.21 (1.45–3.36, p < 0.01), medical school year OR = 2.29, (1.54–3.40, p < 0.01) Barriers Doesn’t perceive CRC as serious health threat OR = 0.7 (0.58–0.94, p = 0.01) Any symptoms OR = 0.71 (0.55–0.91, p = 0.008) Lack of knowledge OR = 0.53 (0.4–0.7, p < 0.01)
Bidouei *et al* [23]	Iran Western Asia	Cross-sectional survey	>40	1,001 478 male	Various methods (FOBT, colonoscopy and sigmoidoscopy)	Descriptive analysis	Self-administered questionnaire or interview/medical students	Fair	Facilitators Knowledge of CRC Family history, employment, education, income had significantly related to knowledge of CRC screening
Chen *et al* [31]	China East Asia	Cross-sectional survey	NA	924	NA	Descriptive analysis	Self-administered questionnaire or interview/medical professionals	Good	NA
Galal *et al* [29]	Saudi Arabia Western Asia Urban and rural	Cross-sectional survey	50–70	884 464 male	FOBT	Descriptive analysis and multivariate	Self-administered questionnaire or interview/health people	Good	Facilitators Gender OR = 0.2 (0.14–0.57), education level OR = 0.3 (0.1–0.8), marital status OR = 0.1 (0.1–0.23)
Hasan *et al* [32]	Pakistan South Asia	Cross-sectional survey	24–60	400 230 male	FOBT, Colonoscopy, sigmoidscopy, FIT	Descriptive analysis	Self-administered questionnaire or interview/health people	Good	Facilitators Knowledge of CRC screening, family history of cancer
Huang.*et al* [17]	Singapore Southeast Asia	Cross-sectional survey	50–75	150 22 male	Various methods (FOBT, colonoscopy and sigmoidoscopy)	Descriptive analysis and multivariate	Self-administered questionnaire or interview/group of non-CRC survivors	Good	Facilitators Household income OR = 3.32 (1.33–8.31, *p* = 0.01), doctors recommendation OR = 7.15 (3–17.7 < 0.001), perceived need to undergo screening OR = 7.1 (3.08–16.4, *p* < 0.78)
Hussain *et al* [33]	Pakistan South Asia	Cross-sectional survey	18–40	302 232 male	NA	Descriptive analysis	Self-administered questionnaire/students of university	Fair	Knowledge
Khayyat and Ibrahim *et al* [30]	Saudi Arabia Western Asia	Cross-sectional survey	>18 <45	313 128 male	NA	Descriptive analysis	Self-administered questionnaire/general population	Fair	Facilitators Awareness of CRC screening ,education, previous knowledge of CRC screening
Ooi *et al* [34]	Malaysia South East Asia	Cross-sectional survey	26–64	197 43 male	NA	Descriptive analysis and multivariate	Self-administered questionnaire/ PCPs working in public clinics	Good	Facilitators Screening being cost-effective OR = 3.3 (1.7–6.6), having adequate resources to do screening OR = 1.9 (1–3.7) significantly related to practice of CRC screening
Alhuzaim *et al* [11]	Saudi Arabia Western Asia	Cross-sectional survey	50–75	925 415 male	FOBT	Descriptive analysis	Self-administered questionnaire and HBM questionnaire/hospital participants	Good	Facilitators Education level had significantly related to knowledge, behaviour and self-efficacy
Yusoff *et al* [35]	Malaysia South East Asia	Cross-sectional survey	NA	1,905 1,022 male	Any of CRC screening	Descriptive analysis	Self-administered questionnaire /primary care clinics with Family Medicine Specialist	Fair	Barriers Embarrassment, uncomfortableness
Thanapirom *et al* [36]	Thailand South East Asia	Cross-sectional survey	NA	387 176 male	FOBT, colonoscopy	Descriptive analysis	Self-administered questionnaire/physicians’ groups, general practitioners, internists, surgeons and other specialists	Fair	Facilitators Gender female, Routinely recommended for CRC screening, work in medical school
Qumseya *et al* [37]	Palestine Western Asia Urban and rural	Cross-sectional survey	50–95	1,352 785 male	Various methods (FOBT, colonoscopy and sigmoidoscopy)	Descriptive analysis and multivariate	Self-administered questionnaire/general population	Good	Willingness Education below secondary school OR = 0.7 (0.53, 0.95, *p* = 0.02), distrust toward western medicine OR = 0.08 (0.04–0.14, *p* < 0.001), religious objection OR = 0.28 (0.09–0.9, *p* = 0.03), embarrassing OR = 0.6 (0.41–0.87, *p* = 0.008), strong fatalistic beliefs OR = 0.69 (0.41–0.87, *p* = 0.02), lack of familiarity with CRC screening OR = 0.55, (0.43–0.7, *p* < 0.001) Urban residence OR = 1.41 (1.03–1.92, *p* = 0.03) Increasing age OR = 1.03 (1.01–1.05, *p* = 0.004)
Tfaily *et al* [38]	Lebanon Western Asia	Cross-sectional survey	25–40	371 161 male	Various methods (FOBT, colonoscopy and sigmoidoscopy)	Descriptive analysis and multivariate	Self-administered questionnaire/patients from hospital	Good	Facilitators Age above 50 years OR = 2.37 (1.36–4.14, *p* = 0.002), regular physician checkups OR = 3.1 (1.88–5.32, *p* = 0 < 0.01), method of awareness about cancer (family doctor) OR = 2.38 (1.2–4.7, *p* = 0.01) related to awareness of CRC screening regular physician checkups significantly, Risk Factor Awareness, related to willingness of CRC screening
Tastan *et al* [39]	Turkey Western Asia	Cross-sectional survey	50–65	160 101 male	15% FOBT 11.3% colonoscopy, 4.4% sigmoidoscopy	Descriptive analysis and multivariate	Self-administered questionnaire and HBM questionnaire/participants from clinic	Good	Facilitators Health motivation is significantly related to education, BMI and exercise. Susceptibility is significantly related to family history of colorectal disease, perceived CRC risk. Severity is significantly related to age, perceived CRC risk, status of information receiving. Barriers BMI, lower education
Taha *et al* [40]	Saudi Arabia Western Asia	Cross-sectional survey	18–50	600 300 male	Various methods (FOBT, colonoscopy and sigmoidoscopy)	Descriptive analysis	A semi-structured questionnaire/participants from different region of country	Good	Facilitators Knowledge of CRC screening is significantly related to knowledge score of CRC disease, history of colon cancer in family, physical recommendation, ever heard or read about CRC screening
Park *et al* [41]	Korea East Asia Urban and rural	Cross-sectional survey	30–74	4,056 1,681 male	46.3% FOBT 34.9% colonoscopy double-contrast barium enema in 10.4%	Descriptive analysis	Self-administered questionnaire /Cancer free man older than 40 years and women older than 30 years	Good	Knowledge
Omran *et al* [42]	Jordan Western Asia Urban, rural	Cross-sectional survey	20–60	160 83 male	NA	Descriptive analysis	Self-administered questionnaire and HBM questionnaire /convenience sample from two hospitals patients and out patients	Good	Barriers Doesn’t have knowledge of CRC screening
Norwati *et al* [43]	Malaysia South East Asia	Cross-sectional study	NA	116 80 male	FOBT	Descriptive analysis	Self-administered questionnaire /primary care clinics with Family Physicians	Fair	Barriers Unavailability of the test, patient in hurry, poor patient awareness
Salimzadeh *et al* [44]	Iran Western Asia	Cross-sectional study	22–75	1,017 423 male	FOBT, colonoscopy	Descriptive analysis	Self-administered questionnaire /Population level screening in which relatives of patients	Good	Knowledge
Ramazani *et al* [45]	Iran Western Asia	Cross-sectional study	>40	480 331 male	FOBT	Descriptive analysis and multivariate	Self-administered questionnaire and HBM/people older than 40 years	Good	Facilitators Digestive problems OR = 2.82 (1.45–5.48), self-efficacy OR = 1.14 (1.04–1.26) are significantly related to CRC screening
Al-Dubai *et al* [46]	Malaysia South East Asia Rural ,semi urban, urban	Cross-sectional study	>30	305 185 male	NA	Descriptive analysis and multivariate	Self-administered questionnaire and HBM Scale/participants from different region of country	Good	Perceived susceptibility Age OR = 2.6 (1.4–4.9) Race OR = 0.2 (0.10–0.7)
Huang *et al* [47]	China East Asia	Population based study	61.70	7,200	FOBT, colonoscopy	Descriptive analysis and multivariate	A population-based telephone survey	Good	Age AOR = 2.01 (0.55–0.7, *p* < 0.001), gender, monthly household income AOR = 0.6 (0.5–0.7, *p* < 0.01) , knowledge of symptoms AOR = 0.62, 0.52–0.74, *p* < 0.001), knowledge of risk factors AOR = 0.46, 0.31–0.68, *p* < 0.001), perceived risk (AOR = 1.32, 1.05–1.65, *p* < 0.5), perceived severity AOR = 2.04 (1.7–2.46, *p* < 0.001), psychological barriers to screening AOR = 0.54 (0.42–0.69, *p* < 0.001), perceived access AOR = 0.55 (0.42–0.69, *p* < 0.001), insurance AOR = 1.22 (1.06–1.41, *p* < 0.01) are significantly related to CRC screening
Dashdebi *et al* [24]	Iran Western Asia	Cross-sectional study	NA	600 289 male	29.9% FOBT	Descriptive analysis and multivariate	Self-administered questionnaire and HBM/clients of private and public laboratories	Good	Facilitators Perceived benefits OR = 0.3 (p < 0.001), self-efficacy OR = 1.6 (p < 0.001), higher education OR = 0.3 (p = 0), information source OR = 1.9 (p = 0.01)
Hilmi *et al* [48]	Malaysia South East Asia	Prospective study	NA	991 459 male	FOBT, colonoscopy, Sigmoidoscopy, barium enema, virtual colonoscopy	Descriptive analysis and multivariate	Self-administered questionnaire and HBM/population with family history of CRC	Good	Facilitators Knowledge of screening Age AOR = 1.69 (1.46–2.65) Gender AOR = 1.59 (1.20–2.11) Ethnicity AOR = 2.5 (1.42–2.94), close family or friends with CRC AOR = 2.67 (1.85–3.88)
Wong *et al* [49]	Singapore South East Asia	Cross-sectional study	>50	1,743 693 male	20.9% FOBT 14% colonoscopy 10.8% sigmoidoscopy	Descriptive analysis and multivariate	Self-administered questionnaire and HBM/population from all household in country	Good	Higher education level
Taheri-Kharameh *et al* [50]	Iran Western Asia	Cross-sectional study	50–70	200 49 male	NA	Descriptive analysis and multivariate	Self-administered questionnaire and HBM /individuals aged 50 and older was recruited from population at outpatient clinics in three teaching hospitals	Good	Gender OR = 3.52 (1.03–11.94) CRC knowledge OR = 2.99 (1.23–5.45) Susceptibility OR = 1.29 (1.86–3.4) Barriers OR = 0.3 (0.21–0.89)
Ng *et al* [51]	Singapore South East Asia	Cross-sectional study	NA	557 241 male	FOBT	Descriptive analysis and multivariate	Self-administered questionnaire and HBM/household units sample	Good	Knowledge score OR = 16.5 (11.2–21.8, p < 0.00) Perceived severity OR = 4.2 (0.4–8.1, p = 0.03) Perceived barrier OR = 7 (2–12, p < 0.01) Perceived benefit OR = 4.8 (1.4–8.1, p < 0.01)
Omran and Ismail *et al* [52]	Jordan Western Asia	Cross-sectional study	>50	200 83 male	FOBT, Colonoscopy	Descriptive analysis	Self-administered questionnaire and HBM /individuals aged 50 and older was recruited from population at two hospitals	Good	Susceptibility Seriousness Health motivation barriers
Bai *et al* [18]	China East Asia	Cross-sectional study	28–70	186 77 male	15.6% colonoscopy	Descriptive analysis and multivariate	Self-administered questionnaire and HBM/people older than 40 years	Good	Perceived Barriers OR = 0.3 (0.12–0.81, *p* = 0.01) Cause to action OR = 3.1 (0.91–10.08, *p* = 0.01)
Al-Thafar *et al* [53]	Saudi Arabia Western Asia Urban and rural	Cross-sectional study	25–55	367 165 male	NA	Descriptive analysis and multivariate	Self-administered questionnaire/teachers	Fair	Higher level of education and age significantly related to knowledge, attitude and practice of CRC screening

a Newcastle–Ottawa Quality Assessment Form for cross-sectional studies

PCPs: primary care physicians; AOR: adjusted odds ratio

**Table 3.** Facilitators and barriers of CRC screening in Asian countries.

Category		Facilitators	Barriers
Patients related factors	Personal factors	Knowledge Attitude Awareness Perceived risk Perceived barriers Perceived severity Perceived seriousness Higher education Family history of cancer Presence of symptoms	Lack of knowledge Lack of awareness Sociodemographic factors High cost, financial problems Fear of result, Fear of procedure, Fear of pain, Embarrassment, shyness Anxiety Unavailability Time constraint No symptom, signs
Health system related factors	Professional factors	Physician recommendation	Physicians recommendations Insufficient guidelines Distrust of screening method

## References

[1] Ferlay J, Shin HR, Bray F (2010). Estimates of worldwide burden of cancer in 2008: GLOBOCAN 2008. Int J Cancer.

[2] Siegel RL, Miller KD, Fedewa SA (2017). Colorectal cancer statistics, 2017. CA Cancer J Clin.

[3] Herbst A, Kolligs FT (2012). Detection of DNA hypermethylation in remote media of patients with colorectal cancer: new biomarkers for colorectal carcinoma. Tumor Biol.

[4] Bray F, Ferlay J, Soerjomataram I (2018). Global cancer statistics 2018: GLOBOCAN estimates of incidence and mortality worldwide for 36 cancers in 185 countries. CA Cancer J Clin.

[5] Sung JJ, Choi SY, Chan FK (2008). Obstacles to colorectal cancer screening in Chinese: a study based on the health belief model. Am Coll Gastroenterol.

[6] Mandel JS, Church TR, Bond JH (2000). The effect of fecal occult-blood screening on the incidence of colorectal cancer. N Engl J Med.

[7] Winawer SJ, Zauber AG, Ho MN (1993). Prevention of colorectal cancer by colonoscopic polypectomy. N Engl J Med.

[8] World Health Organization (2009). Towards a Strategy for Cancer Control in the Eastern Mediterranean Region.

[9] Levin B, Lieberman DA, McFarland B (2008). Screening and surveillance for the early detection of colorectal cancer and adenomatous polyps, 2008: a joint guideline from the American Cancer Society, the US Multi-Society Task Force on Colorectal Cancer, and the American College of Radiology. Gastroenterology.

[10] Rafiemanesh H, Mohammadian-Hafshejani A, Ghoncheh M (2016). Incidence and mortality of colorectal cancer and relationships with the human development index across the world. Asian Pac J Cancer Prev.

[11] Alhuzaim W, Alosaimi M, Almesfer AM (2020). Saudi patients' knowledge, behavior, beliefs, self-efficacy and barriers regarding colorectal cancer screening. Int J Pharm Res Allied Sci.

[12] Al Abdouli L, Dalmook H, Akram Abdo M (2018). Colorectal cancer risk awareness and screening uptake among adults in the United Arab Emirates. Asian Pac J Cancer Prev.

[13] Ghoncheh M, Mohammadian M, Mohammadian-Hafshejani A (2016). The incidence and mortality of colorectal cancer and its relationship with the human development index in Asia. Ann Glob Health.

[14] Al-Hajeili M, Abdulwassi HK, Alshadadi F (2019). Assessing knowledge on preventive colorectal cancer screening in Saudi Arabia: a cross-sectional study. J Family Med Prim Care.

[15] Bai Y, Wong CL, He X (2020). Effectiveness of tailored communication intervention in increasing colonoscopy screening rates amongst first-degree relatives of individuals with colorectal cancer: a systematic review and meta-analysis. Int J Nurs Stud.

[16] Almasi Z, Mohammadian-Hafshejani A, Salehiniya H (2016). Incidence, mortality, and epidemiological aspects of cancers in Iran; differences with the world data. J BUON.

[17] Huang Y, Soon YY, Ngo LP (2019). A cross-sectional study of knowledge, attitude and barriers to colorectal cancer screening among cancer survivors. Asian Pac J Cancer Prev.

[18] Bai Y, Wong CL, Peng X (2020). Colonoscopy screening behaviour and associated factors amongst first-degree relatives of people with colorectal cancer in china: testing the health belief model using a cross-sectional design. Int J Environ Res Public Health.

[19] Mozafar Saadati H, Khodamoradi F, Salehiniya H (2020). Associated factors of survival rate and screening for colorectal cancer in Iran: a systematic review. J Gastrointest Cancer.

[20] Alduraywish SA, Altamimi LA, Almajed AA (2020). Barriers of colorectal cancer screening test among adults in the Saudi Population: a cross-sectional study. Prev Med Rep.

[21] Almadi MA, Mosli MH, Bohlega MS (2015). Effect of public knowledge, attitudes, and behavior on willingness to undergo colorectal cancer screening using the health belief model. Saudi J Gastroenterol.

[22] Al-Naggar RA, Al-Kubaisy W, Yap BW (2015). Attitudes towards colorectal cancer (CRC) and CRC screening tests among elderly Malay patients. Asian Pac J Cancer Prev.

[23] Bidouei F, Abdolhosseini S, Jafarzadeh N (2014). Knowledge and perception toward colorectal cancer screening in east of Iran. Int J Health Policy Manag.

[24] Dashdebi KG, Noroozi A, Tahmasebi R (2016). Factors predicting fecal occult blood testing among residents of Bushehr, Iran, based on the health belief model. Asian Pac J Cancer Prev.

[25] Berkowitz Z, Hawkins NA, Peipins LA (2008). Beliefs, risk perceptions, and gaps in knowledge as barriers to colorectal cancer screening in older adults. J Am Geriatr Soc.

[26] Sirriyeh R, Lawton R, Gardner P (2012). Reviewing studies with diverse designs: the development and evaluation of a new tool. J Eval Clin Pract.

[27] Almadi MA, Alghamdi F (2019). The gap between knowledge and undergoing colorectal cancer screening using the Health Belief Model: a national survey. Saudi J Gastroenterol.

[28] Althobaiti A, Jradi H (2019). Knowledge, attitude, and perceived barriers regarding colorectal cancer screening practices and risk factors among medical students in Saudi Arabia. BMC Med Educ.

[29] Galal YS, Amin TT, Alarfaj AK (2016). Colon cancer among older saudis: awareness of risk factors and early signs, and perceived barriers to screening. Asian Pac J Cancer Prev.

[30] Khayyat YM, Ibrahim EM (2014). Public awareness of colon cancer screening among the general population: a study from the Western Region of Saudi Arabia. Qatar Med J.

[31] Chen YS, Xu SX, Ding YB (2013). Colorectal cancer screening in high-risk populations: a survey of cognition among medical professionals in Jiangsu, China. Asian Pac J Cancer Prev.

[32] Hasan F, Shah SMM, Munaf M (2017). Barriers to colorectal cancer screening in Pakistan. Cureus.

[33] Hussain I, Majeed A, Rasool MF (2020). Knowledge, Attitude, preventive practices and perceived barriers to screening about colorectal cancer among university students of newly merged district, Kpk, Pakistan – a cross-sectional study. J Oncol Pharm Pract.

[34] Ooi CY, Hanafi NS, Liew SM (2019). Knowledge and practice of colorectal cancer screening in an urban setting: cross-sectional survey of primary care physicians in government clinics in Malaysia. Singapore Med J.

[35] Yusoff HM, Daud N, Noor NM (2012). Participation and barriers to colorectal cancer screening in Malaysia. Asian Pac J Cancer Prev.

[36] Thanapirom K, Treeprasertsuk S, Rerknimitr R (2012). Awareness of colorectal cancer screening in primary care physicians. J Med Assoc Thailand.

[37] Qumseya BJ, Tayem YI, Dasa OY (2014). Barriers to colorectal cancer screening in palestine: a national study in a medically underserved population. Clin Gastroenterol Hepatol.

[38] Tfaily MA, Naamani D, Kassir A (2019). Awareness of colorectal cancer and attitudes towards its screening guidelines in Lebanon. Ann Glob Health.

[39] Tastan S, Andsoy II, Iyigun E (2013). Evaluation of the knowledge, behavior and health beliefs of individuals over 50 regarding colorectal cancer screening. Asian Pac J Cancer Prev.

[40] Taha I (2019). Assessment of knowledge about colorectal cancer in Saudi Arabia. Indo Am J Pharm Sci.

[41] Park B, Lee H-Y, Choi KS (2011). Cancer Screening in Korea, 2010: Results from the Korean National Cancer Screening Survey. Asian Pac J Cancer Prev.

[42] Omran S, Barakat H, Muliira JK (2015). Knowledge, experiences, and barriers to colorectal cancer screening: a survey of health care providers working in primary care settings. J Cancer Educ.

[43] Norwati D, Harmy MY, Norhayati MN (2014). Colorectal cancer screening practices of primary care providers: results of a national survey in Malaysia. Asian Pac J Cancer Prev.

[44] Salimzadeh H, Bishehsari F, Delavari A (2016). Cancer risk awareness and screening uptake in individuals at higher risk for colon cancer: a cross-sectional study. BMJ Open.

[45] Ramazani AA, Norozi E, AmirabadiZadeh H (2020). Predictors of Colorectal Cancer Screening Participation in Southern Khorasan (Iran). J Gastrointest Cancer.

[46] Al-Dubai SAR, Ganasegeran K, Alabsi AM (2013). Exploration of risk taking behaviors and perceived susceptibility of colorectal cancer among Malaysian adults: a community based cross-sectional study. BMC Public Health.

[47] Huang J, Choi P, Pang TWY (2020). Factors associated with participation in colorectal cancer screening: a population-based study of 7200 individuals. Eur J Cancer Care.

[48] Hilmi I, Hartono JL, Goh K (2010). Negative perception in those at highest risk--potential challenges in colorectal cancer screening in an urban asian population. Asian Pac J Cancer Prev.

[49] Wong RK, Wong ML, Chan YH (2013). Gender differences in predictors of colorectal cancer screening uptake: a national cross sectional study based on the health belief model. BMC Public Health.

[50] Taheri-Kharameh Z, Noorizadeh F, Sangy S (2016). Factors associated with adherence to colorectal cancer screening among moderate risk individuals in Iran. Asian Pac J Cancer Prev.

[51] Ng EST, Tan CH, Teo DC (2007). Knowledge and perceptions regarding colorectal cancer screening among Chinese – a community-based survey in Singapore. Prev Med.

[52] Omran S, Ismail AA (2010). Knowlednge and beliefs of Jordanians toward colorectal cancer screening. Cancer Nurs.

[53] Al-Thafar AK, Al-Naim AF, Albges DS (2017). Knowledge attitude and practice of colorectal cancer among school teachers in Al-Ahsa Saudi Arabia. Asian Pac J Cancer Prev.

[54] Waller J, Macedo A, Wagner C (2012). Communication about colorectal cancer screening in Britain: public preferences for an expert opinion. Br J Cancer.

[55] Klabunde CN, Schenck AP, Davis WW (2006). Barriers to colorectal cancer screening among medicare consumers. Am J Prev Med.

